# Vasopressin improves outcome in out-of-hospital cardiopulmonary resuscitation of ventricular fibrillation and pulseless ventricular tachycardia: a observational cohort study

**DOI:** 10.1186/cc3967

**Published:** 2006-01-06

**Authors:** Stefek Grmec, Stefan Mally

**Affiliations:** 1 Assistant Professor, Head of the Department, Centre for Emergency Medicine Maribor, University of Maribor, Maribor, Slovenia; 2 Medical Doctor, Centre for Emergency Medicine Maribor, Maribor, Slovenia

## Abstract

**Introduction:**

An increasing body of evidence from laboratory and clinical studies suggests that vasopressin may represent a promising alternative vasopressor for use during cardiac arrest and resuscitation. Current guidelines for cardiopulmonary resuscitation recommend the use of adrenaline (epinephrine), with vasopressin considered only as a secondary option because of limited clinical data.

**Method:**

The present study was conducted in a prehospital setting and included patients with ventricular fibrillation or pulseless ventricular tachycardia undergoing one of three treatments: group I patients received only adrenaline 1 mg every 3 minutes; group II patients received one intravenous dose of arginine vasopressine (40 IU) after three doses of 1 mg epinephrine; and patients in group III received vasopressin 40 IU as first-line therapy. The cause of cardiac arrest (myocardial infarction or other cause) was established for each patient in hospital.

**Results:**

A total of 109 patients who suffered nontraumatic cardiac arrest were included in the study. The rates of restoration of spontaneous circulation and subsequent hospital admission were higher in vasopressin-treated groups (23/53 [45%] in group I, 19/31 [61%] in group II and 17/27 [63%] in group III). There were also higher 24-hour survival rates among vasopressin-treated patients (*P *< 0.05), and more vasopressin-treated patients were discharged from hospital (10/51 [20%] in group I, 8/31 [26%] in group II and 7/27 [26%] group III; *P *= 0.21). Especially in the subgroup of patients with myocardial infarction as the underlying cause of cardiac arrest, the hospital discharge rate was significantly higher in vasopressin-treated patients (*P *< 0.05). Among patients who were discharged from hospital, we found no significant differences in neurological status between groups.

**Conclusion:**

The greater 24-hour survival rate in vasopressin-treated patients suggests that consideration of combined vasopressin and adrenaline is warranted for the treatment of refractory ventricular fibrillation or pulseless ventricular tachycardia. This is especially the case for those patients with myocardial infarction, for whom vasopressin treatment is also associated with a higher hospital discharge rate.

## Introduction

Survival after cardiopulmonary resuscitation (CPR) with adrenaline (epinephrine) therapy is disappointing [1,2]. The use of adrenaline is associated with increased myocardial oxygen consumption, ventricular arrhythmias and myocardial dysfunction during the period following resuscitation [3-5]. In the American Heart Association 2000 Guidelines and in the Emergency Cardiovascular Care Guidelines 2000 for Cardiopulmonary Resuscitation and Emergency Cardiovascular Care, vasopressin is considered a secondary alternative to adrenaline in the treatment of unstable ventricular tachycardia (VT) and ventricular fibrillation (VF)[6,7]. During CPR it significantly improves total cerebral and left myocardial blood flow, and it causes a sustained increase in mean arterial blood pressure as compared with maximal doses of adrenaline [8-14]. However, some clinical studies yielded contrasting findings [15-19]. Moreover, clinical experience with vasopressin as an alternative to adrenaline for vasopressor therapy in CPR is limited [6,7,20-25].

We conducted a clinical investigation to assess the effect of vasopressin on outcome in out-of-hospital CPR for VF and pulseless VT. Our hypothesis was that vasopressin improves outcome in VF/VT cardiac arrest, especially in patients with acute myocardial infarction (AMI).

## Materials and methods

We undertook a prospective observational cohort study, with a retrospective control group, in a prehospital setting, after approval had been granted by the ethical review board of the Ministry of Health of Slovenia. The study community, in the region surrounding the city of Maribor in Slovenia, includes a population of 190,000, and approximately 90 resuscitations are attempted per year. The initial response to cardiac arrest is by emergency doctors (prehospital emergency unit). Basic and advanced cardiac life support are provided by emergency doctors before the patient's arrival to the hospital, applying a regional protocol that incorporates European Resuscitatation Council standards, guidelines and clinical algorithms for CPR.

This study included only victims of cardiac arrest with registered initial VF or pulseless VT. We compared three treatments. Group I patients received only adrenaline 1 mg every three minutes (data were collected from February 1998 to October 2000). In group II patients received one intravenous dose of 40 IU arginine vasopressin (Pitressin™; Goldshield Pharmaceuticals, Croydon, UK) after three doses of 1 mg adrenaline (data were collected prospectively from November 2000 to October 2003). Finally, group III patients received arginine vasopressin 40 IU as first-line therapy (data were collected prospectively from November 2003 to December 2004). If there was no return of pulse after vasopressin, patients received adrenaline 1 mg every three minutes during CPR. Demographic and clinical characteristics of the patients were similar in the three groups.

Exclusion criteria were successful defibrillation without administration of a vasopressor, age under 18 years, documented terminal illness, traumatic cardiac arrest, severe hypothermia (<30°C), pulseless electrical activity or asystole as initial rhythm at arrival, and inability to gain intravenous access. All drugs were injected exclusively intravenously, followed by 20 ml normal saline.

The causes of cardiac arrest were divided into AMI and other. The criteria used for diagnosis of AMI and for primary arrhythmia are consistent with current standards (for instance, those of the World Health Organization, and the Joint European Society of Cardiology/American College of Cardiology Committee) [26-29]. In the group of 'other' causes of cardiac arrest, we included submersion, respiratory causes, intoxication, electrolytic and endocrinologic disorders, and unknown aetiology. Diagnoses were confirmed in the intensive care unit or, for those patients who died at the scene, at autopsy.

The data regarding CPR in the prehospital setting were collected in accordance with directions presented by the ILCOR (International Liasion Committee on Resuscitation) Task Force on Cardiac Arrest and Cardiopulmonary Resuscitation Outcomes [30].

Data are expressed as mean ± standard deviation or number (%). Comparisons between groups were performed using Fisher's exact test for categorical data and Wilcoxon's rank-sum test for numerical data. Bonferroni correction was applied for multiple comparison. The null hypothesis was considered to be rejected at *P *values less than 0.05. Multiple logistic regression analysis was done to examine the relationship between survival and application of vasopressin, adjusting for age, sex, time elapsed before initiation of CPR, time of resuscitation by the medical team, witnessed arrest, and basic life support by bystanders. The results were expressed as odds ratio (95% confidence interval). All analyses were conducted using SPSS version 12.0 software (SPSS, Inc., Chicago, IL, USA).

## Results

The total numbers of episodes of cardiac arrest for the three study periods (for example, groups I, II and III), the number of resuscitation attempts along with the specific rhythms and outcomes, and various other CPR variables are summarized in Table 1.

We retrospectively studied 51 adult patients in nontraumatic, out-of-hospital VF/VT cardiac arrest who received only adrenaline 1 mg every three minutes during CPR (group I). The average dose of adrenaline was 6.3 ± 3.5 mg (range 1–16 mg; Table 2). This value was higher than the average dose of adrenaline in the vasopressin groups (groups II and III; *P *< 0.05). We also prospectively studied 31 patients in VF/VT cardiac arrest who received vasopressin after three doses of 1 mg adrenaline (group II), and 27 patients who received vasopressin as the first-line therapy (group III). There were no statistically significant differences in sex, age, time elapsed before initiation of CPR, suspected cause of cardiac arrest, witnessed arrest, and bystander basic life support between the three groups (Table 2). The time to resuscitation by the medical team was significantly longer in patients in the adrenaline-only group than in the vasopressin groups (*P *< 0.05). The rate of restoration of spontaneous circulation (ROSC) with hospital admission, and the 24-hour survival rate were significantly higher among patients in the vasopressin groups (*P *< 0.05); rates were similar between the two vasopressin groups (*P *= 0.79; Table 2).

With respect to resuscitation outcomes, in group II (three doses of adrenaline first, followed by vasopressin; 113 patients with initial VF/VT rhythm) 29 patients were resuscitated after adrenaline only (29/113 [26%]) and 11 patients were resuscitated after vasopressin was given, without additional doses of adrenaline (11/31 [36%]). In group III (vasopressin as first-line therapy; 38 patients with initial VF/VT rhythm) 10 patients were resuscitated after a single dose of vasopressin (10/38 [27%]). Demographic characteristics and causes of cardiac arrest are summarized in Table 2.

More patients treated with vasopressin (but not significantly more) were discharged from hospital (*P *= 0.21). There were no significant differences in neurological status between the groups at discharge. For group I Cerebral Performance Category (CPC) values were as follows: six out of ten patients had CPC 1 or 2; three out of ten had CPC 3 or 4; and one out of ten had CPC 5. For group II the values were as follows: five out of eight patients had CPC 1 or 2; and three out of eight had CPC 3 or 4. Finally, for group III the CPC values were as follows: four out of seven patients had CPC 1 or 2; and three out of seven had CPC 3 or 4.

When adjusting for differences in age, sex, basic life support from bystanders, time elapsed before initiation of CPR, witnessed arrest, response time, and administration of amiodarone and bicarbonate, the odds ratio for ROSC among patients who received vasopressin (groups II and III) versus the adrenaline group (group I) was 3.1 (95% confidence interval 1.7–8.3; *P *< 0.01). With the same adjustment as for ROSC, the odds ratio for survival of the first 24 hour survival among patients who received vasopressin versus the adrenaline group was 3.8 (95% confidence interval 1.5–9.1; *P *< 0.01).

Rates of ROSC with admission to hospital and hospital discharge in patients with AMI were significantly higher in the vasopressin groups than in the adrenaline group (discharge: four out of 32 patients [12.5%] in group I; five out of 20 patients [25%] in group II; five out of 18 patients [28%] in group III; *P *< 0.05). The adjusted odds ratio for ROSC with admission to hospital among patients with AMI in the vasopressin groups versus the adrenaline group was 2.8 (95% confidence interval 1.4–4.8; *P *< 0.01). The adjusted odds ratio for surviving to hospital discharge among patients with AMI in the vasopressin groups versus the adrenaline group was 2.9 (95% confidence interval 1.1–5.3; *P *= 0.01). There was no significant difference between the groups in ROSC rate when patients with primary arrythmia were compared with patients with other causes of cardiac arrest (Table 3).

## Discussion

For patients in cardiac arrest with refractory VF or pulseless VT after defibrillation, administration of a vasopressor is intended to improve myocardial and cerebral perfusion. It should not increase myocardial oxygen demand or promote arrythmias [22,23]. Adrenaline increases myocardial oxygen demand and consumption [2-4], decreases myocardial ATP with proarrhythmic effects [2], and increases myocardial lactate level [3-5,23]. Prehospital administration of adrenaline appears to be of little value in increasing rates of survival to discharge, and the cumulative dose of adrenaline is an independent predictor of poor neurological outcome [1,9,24]. Adrenaline increases intrapulmonary shunting by 30% and decreases arterial oxygen saturation [4]. It also significantly increases the severity of post-resuscitation myocardial dysfunction, and consequently it decreases post-resuscitation survival [23,25].

Vasopressin is an attractive alternative to adrenaline during CPR because it significantly improves cerebral and myocardial blood flow by virtue of its nitric oxide vasodilatatory effect [8,11,12,14]. The potential benefits of vasopressin in CPR arise primarily from its ability to stimulate the contraction of vascular smooth muscle, resulting in peripheral vasoconstriction and increased blood pressure. Unlike adrenaline, vasopressin has no β-adrenergic effects and is resistant to the effects of acidosis [16,23]. It does not decrease myocardial ATP level and does not increase myocardial lactate level [9,10,12,23].

Comparing the groups in our trial, significant differences were found between vasopressin groups (groups II and III) and the adrenaline group (group I) in the rate of ROSC with hospitalization and in 24-hour survival rate. In the study there were no significant differences in rates of hospital discharge between vasopressin groups and adrenaline group, as was reported previously [15,17-19]. Lindner and coworkers [17] reported that a significantly larger proportion of patients treated with vasopressin were resuscitated and survived 24 hours as compared with those treated with adrenaline. Stiell and coworkers [15] observed no difference between adrenaline and vasopressin groups in survival rates at 1 hour and 30 days. Several differences between these two studies may account for their results. Vasopressin was administered much later in the study by Lindner and coworkers than in that by Stiell and colleagues. Compared with adrenaline, vasopressin exerts greater vasoconstriction in hypoxic and acidotic conditions [13], and so the rapid response and early treatment in the study by Stiell and colleagues may explain the lack of difference observed between vasopressin and adrenaline [23]. Vasopressin improved perfusion pressures during CPR in patients with VF/VT in a trial conducted by Wenzel and coworkers [18], but it did not improved the outcome. In that trial there was no difference in findings between vasopressin groups. This observation may indicate that the interactions between adrenaline and vasopressin improve ROSC and short-term survival in VF/VT arrest. In the present study we also showed that the sequence of vasopressin administered (for example, initially or after adrenaline) was not important; what was important was combined therapy with the two drugs. This findings suggests that the presence of one of these drugs may enhance the effects of the other.

In patients with myocardial infarction we found significantly higher rates of ROSC and hospital discharge in groups treated with vasopressin than in the adrenaline group. This observation has potentially important consequence for the treatment of VF/VT cardiac arrest in the prehospital setting. Our findings strongly support combined administration of vasopressin and adrenaline during CPR among patients in VF/VT arrest caused by myocardial infarction.

Our study has some important limitations. This observational study was limited by the small number of patients included, and our findings in patients with AMI require confirmation in a larger multicentre clinical trial. Indeed, the ideal comparison between the three groups would be performed within the context of a randomized controlled clinical trial; however, such a 'perfect' study could may be considered unethical because, in our view, it is unacceptable to withhold vasopressin when it is available. We are aware that the nonrandomized setting in which our study was conducted dilutes the value of our conclusions but, in a field in which there are few clinical investigations, we believe that the study provides important additional data that may help to improve outcomes in patients with cardiac arrest.

## Conclusion

The better results in vasopressin-treated groups suggest that there is an indication for combined use of vasopressors (such as vasopressin and adrenaline) in out-of-hospital resuscitation for refractory VF/VT cardiac arrest, especially in patients with myocardial infarction.

## Key messages

• 	The rate of ROSC with hospital admission and the 24-hour survival rate were higher among patients administered vasopressin than in those treated with adrenaline, irrespective of the cause of cardiac arrest.

• 	The rate of hospital discharge was significantly higher in patients with myocardial infarction as the cause of cardiac arrest, when they were treated with vasopressin.

## Abbreviations

AMI = acute myocardial infarction; CPC = Cerebral Performance Category; CPR = cardiopulmonary resuscitation; ROSC = restoration of spontaneous circulation; VF = ventricular fibrillation; VT = ventricular tachycardia.

## Competing interests

The authors declare that they have no competing interests.

## Authors' contributions

GS participated in conceiving and designing the study, performed the statistical analysis, and helped to draft the manuscript. MS participated in designing the study and drafted the manuscript. Both authors read and approved the final manuscript.

## Figures and Tables

**Table 1:** Utstein reporting for CPR data for three different periods (groups)

CPR data	Period I (February 1998 to October 2000)	Period II (November 2000 to October 2003)	Period III (November 2003 to December 2004)
Resuscitation attempted (*n*)	165	274	91

First monitored rhythm (*n*)			
Shockable:	60	113	38
Included in study	51 (group I)^a^	31 (group II)^b^	27 (group III)^c^
VF	55	99	34
VT	5	14	4
Nonshockable:	105	161	53
Asystole	83	121	34
PEA	22	40	19

Location of arrest (*n*)			
Home	92	166	52
Public place	46	79	31
Other	27	29	8

Arrest witnessed (*n*)	102	176	61
By layperson/bystander	85	153	50
By health care personnel	17	23	11
Arrest not witnessed (*n*)	63	98	30

Etiology (*n*)			
Presumed cardiac	98	161	62
Trauma	5	8	4
Submersion	4	6	3
Respiratory	19	34	18
Other noncardiac	18	38	4
Unknown	21	27	0

Outcome (*n*)			
Any ROSC	114	185	53
Survived event	74	138	44
Discharged alive	34	63	18

Utstein recommendations on CPR data reporting are summarized by Jacobs and coworkers [30]. ^a^Nine patients were excluded (9/60 [15%]) from the study because of successful resuscitation after the first series of shocks (200, 200, 360 J). ^b^Eighty-two patients were excluded: 15/113 patients (13%) were excluded from the study because of successful resuscitation after the first series of shocks (200, 200, 360 J); an additional 29/113 patients (26%) were excluded after pulse was restored during administration of the three initial doses of adrenaline (up to 3 mg); and a further 38 patients were excluded because vasopressin was not available during CPR. ^c^Eleven patients were excluded: 5/38 (13%) patients with pulse after the first series of shocks (200, 200, 360 J) and 6/38 (16%) patients receiving vasopressin after adrenaline was administered. CPR, cardiopulmonary resuscitation; PEA, pulseless electrical activity; ROSC, restoration of spontaneous circulation; VF, ventricular fibrillation; VT, ventricular tachycardia.

**Table 2:** Characteristics and survival outcomes in three treatment groups of patients with VF/VT cardiac arrest

Characteristics/survival outcomes	Treatment group		
	
	Adrenaline only (group I)	Vasopressin after adrenaline (group II)	Vasopressin initially (group III)
Sex (male/female; *n*)^a^	29/22	17/14	15/12
Age^b^	61.3 ± 15.3	59.2 ± 13.3	60.3 ± 17.3
Time to initiation of CPR (min)^b^	6.3 ± 3.5	7.2 ± 3.2	6.3 ± 2.9
Average dose of adrenaline (mg)^b^	6.3 ± 3.5*	4.5 ± 2.1	3.1 ± 1.6
Amiodarone (*n *[%])^a^	9/51 (18%)*	21/31 (68%)	17/27 (63%)
Lidocaine (*n *[%])^a^	24/51 (47%)*	5/31 (16%)	1/27 (4%)
Bicarbonate (*n *[%])^a^	23/51 (45%)	8/31 (26%)	6/27 (22%)
Atropine (*n *[%])^a^	10/51 (20%)	4/31 (13%)	3/27 (11%)
ROSC with hospitalization (*n *[%])^a^	23/51 (45%)*	19/31 (61%)	17/27 (63%)
24 hour survival (*n *[%])^a^	15/51 (29%)*	15/31 (48%)	14/27 (51%)
Hospital discharge (*n *[%])^a^	10/51 (20%)	8/31 (26%)	7/27 (26%)
Resuscitation by medical team (min)^b^	28.1 ± 11.8*	18.8 ± 9.3	17.4 ± 8.4
Witnessed arrest (*n *[%])^a^	32/51 (62%)	17/31 (55%)	16/27 (59%)
Cause of arrest (AMI/primary arrhythmia/other; *n*)^a^	32/6/13	20/4/7	18/3/6

^a^By Fisher exact test; ^b^by Wilcoxon rank-sum test. *P < 0.05 versus the other two groups. AMI, acute myocardial infarction; CPR, cardiopulmonary resuscitation; ROSC, restoration of spontaneous circulation; VF, ventricular fibrillation; VT, ventricular tachycardia.

**Table 3:** Rates of ROSC and hospital discharge among different causes of VF/VT cardiac arrest

ROSC and discharge rates	Adrenaline only (group I)	Vasopressin after adrenaline (group II)	Vasopressin initially (group III)
Myocardial infarction (*n *[%])			
ROSC	11/32 (34%)*	12/20 (60%)	11/18 (61%)
Discharge	4/32 (12%)*	5/20 (25%)	5/18 (28%)

Primary arrhythmia (*n *[%])			
ROSC	4/6 (66%)	3/4 (75%)	2/3 (67%)
Discharge	3/6 (50%)	2/4 (50%)	1/3 (33%)

Other causes (*n *[%])			
ROSC	8/13 (61%)	4/7 (57%)	4/6 (67%)
Discharge	3/13 (23%)	1/7 (14%)	1/6 (17%)

*P < 0.05 versus the two other groups. ROSC, restoration of spontaneous circulation; VF, ventricular fibrillation; VT, ventricular tachycardia.
